# The association between duration of muscle exercise and psychological symptoms is higher in boys than in girls: A cross-sectional study based on Chinese college students during COVID-19

**DOI:** 10.3389/fped.2022.1036176

**Published:** 2022-11-24

**Authors:** Yu Ouyang, Yu Hong, Jun Cheng, Hongmin Cao

**Affiliations:** ^1^ School of Physical Education & Health, Nangchang Institute of Science & Technology, Jiangxi, China; ^2^ School of Physical Education, Chizhou University, Chizhou, China

**Keywords:** muscle exercise, psychological symptoms, college students, association, China

## Abstract

**Objective:**

The COVID-19 pandemic has had many negative effects on the physical and mental health of college students. Although many studies have analyzed the association between muscular fitness and psychological symptoms in children and adolescents, research during the COVID-19 pandemic is limited. Our study focused on analyzing the association between duration of muscle exercise and psychological symptoms among Chinese college students during the COVID-19 pandemic.

**Method:**

A four-stage stratified whole-group sampling method was used to investigate basic demographic information, duration of muscle exercise and psychological symptoms in 5,559 college students aged 19–22 years in China. Chi-square test (categorical variables) and one-way ANOVA (continuous variables) were used to compare the psychological symptoms of college students with different durations of muscle exercise. Logistic regression analysis was used to analyze the association between duration of muscle exercise and psychological symptoms.

**Result:**

The detection rate of psychological symptoms among Chinese college students was 9.0%; the detection rate was 10.7% for boys and 7.6% for girls. The proportions of duration of muscle exercise at <30 min/d, 30–60 min/d, and >60 min/d were 75.0%, 20.4%, and 4.6%, respectively. After adjusting for relevant confounding variables, taking Chinese college students with duration of muscle exercise >60 min/d as the reference group, duration of muscle exercise <30 min/d was positively correlated with the occurrence of psychological symptoms (*OR*: 4.19, 95%CI: 1.82, 9.61) (*P *< 0.001). In emotional symptoms (*OR*: 4.56, 95%CI: 1.99, 10.44), behavioral symptoms (*OR*: 3.44, 95%CI: 1.79, 6.60), social adaptation difficulties (*OR*: 3.04, 95%CI: 1.62, 5.68) dimensions, there is also a positive correlation (*P* < 0.01).

**Conclusions:**

The negative association between duration of muscle exercise and psychological symptoms among Chinese college students also suggests that longer duration of muscle exercise among college students is associated with a lower prevalence of psychological symptoms. The association between duration of muscle exercise and psychological symptoms was higher in boys compared to girls.

## Introduction

As a main component of physical fitness, muscular strength has been increasingly recognized in the pathogenesis and prevention of chronic disease (1, 2). Whereas loss of muscle strength is strongly related to functional limitations and physical disability (3, 4). Meanwhile, youth with low muscular fitness are at increased risk of maintaining a low muscular fitness level into adulthood (5). Moreover, several reports suggested that muscular strength is independently associated with death even after controlling many confounding factors (6, 7). Narrative reviews also concluded that muscular strength can be considered as a strong predictor of mortality (8, 9).

Adolescence is a period of rapid social, emotional, and cognitive development and key life transitions. A recently editorial comment suggested that, being at an age of uncertainty, mental health in young people is undoubtedly concerning (10). A systematic review including 98,044 children aged 6–12 years found out that most mental disorders are more prevalent in adolescence, a stage that lasts up to age 24 years, and the pooled prevalence for suicidal ideation reached 7.5% (95% *CI* 5.9–9.6) (11).

Animal experimental studies suggested that exercise may attenuate depression through target in the skeletal muscle (12). Growing evidence also suggested the inverse relationship between muscular strength and mental health among middle and elder adults (13, 14), including longitudinal cohort study estimating the bidirectional associations between them (15), prospective cohort study exploring the ability of muscle strength to predict depressive symptoms (16). However, limited study focus on young people especially on college students. To our best knowledge, we find one study indicated that handgrip strength is inversely and independently related to the risk of depressive symptoms among Chinese girls college, while the Chinese boys college were not involved (17).

During the COVID-19 pandemic, college students around the world including China, experienced disrupted schooling and social isolation, and meta-analysis reported higher prevalence of depression (39%) or anxiety (36%) than before the outbreak (18). The present study aimed to estimate the associations between muscular strength and psychological symptom among Chinese college students.

## Materials and methods

### Data sources and participants recruitment

From September to December 2021, this study adopts the method of stratified cluster sampling to sample the tested college students. The sampling process was divided into four stages. In the first stage, on the basis of China's overall geographic planning, taking into account the geographic locations of different cities, Anhui, Jiangxi, and Xinjiang were finally selected as sample provinces for research. In the second stage, each province selects a provincial capital city university with a higher level of economic development according to the difference in economic level, and then selects another university in a region with a lower level of economic development as the survey school. In the third stage, 1 college in each university was randomly selected. In the fourth stage, a questionnaire survey was administered to all college students enrolled in the sampled colleges who met the inclusion criteria. The specific inclusion criteria were: (1) enrolled college students; (2) no major physical or mental illness; and (3) informed consent and voluntary acceptance of the study. Finally, a total of 5,781 enrolled college students from six colleges were selected for our study, and after excluding questionnaires with incomplete demographic important information after the survey, 5,559 valid questionnaires were finally returned, with a valid response rate of 96.2%.

Written informed consent for our study was obtained from the students themselves, their guardians, and the school prior to the investigation, and the college students gave informed and voluntary consent to the investigation and signed a written informed consent form prior to the investigation. Our study was approved by the Human Ethics Committee of Chizhou University (20210403).

### Basic information and covariates

The information investigated in our study included province, age, gender, grade, class, parental education, socioeconomic status (SES), sleep duration, body mass index (BMI), and moderate and vigorous physical activity (MVPA). Some of the surveyed indicators were adjusted as covariates in the regression analysis. Parents' education level was divided into primary school and below, secondary and high school, and college degree and above. SES was evaluated comprehensively according to the subjects' parents’ occupation, parents' educational background and household electrical appliances. Sleep duration was divided into two groups of ≥8 h/d and <8 h/d. BMI was calculated based on height and weight obtained from the test, and the formula was weight (kg)/height (m)^2^. MVPA was obtained based on the average daily duration of subjects' participation in moderate-to-vigorous physical activity in the past 7 days. The physical activity questionnaire was investigated using the physical activity section of the Chinese Student Physical Fitness Survey questionnaire. Age, father's education level, mother's education level, SES, sleep duration, BMI, and MVPA were adjusted as covariates in this study according to the different models. The questionnaire has good reliability and validity.

### Duration of muscle exercise

Our study investigated the duration of muscle exercise based on the entries of the Chinese Student Physical Fitness Survey. The Chinese Student Physical Fitness Survey is a nationwide student physical fitness monitoring program conducted by the Chinese government to further promote and improve the physical fitness of children and adolescents (19). The program is jointly implemented by the Ministry of Education, the State General Administration of Sports and other departments, and physical fitness tests are conducted once every five years on selected school and college students across China. There are more than 30 test indicators, including physical form, physical function, physical fitness, blood pressure, dental caries and other health checks. A questionnaire was also included, covering lifestyle, physical exercise, physical activity, sleep quality, and mental health status. The questionnaire has good reliability and validity.

The specific questions used in our study were: what was the average daily duration of muscle strength exercise (e.g., squats, push-ups, pull-ups, use of dumbbells, exercise with fitness equipment) during the past 7 days? The options were divided into three categories: <30 min/d, 30–60 min/d, and >60 min/d.

### Psychological symptoms

Our survey of psychological symptoms among Chinese college students was conducted using the multidimensional sub-health questionnaire of adolescents (MSQA). This scale has been used to test several studies of psychological symptoms in Chinese children and adolescents and has good reliability and validity (20).

Our study was conducted using a short 15-item questionnaire, which also has good reliability (21). The questionnaire consisted of three dimensions: emotional symptoms, behavioral symptoms, social adaptation difficulties, and psychological symptoms, which reflect the overall psychological status. The emotional symptoms consisted of 7 items, such as “often feel nervous” and “often feel restless and distracted”. The behavioral symptoms consist of 4 items, such as “I always feel that people are against me” and “I am often angry about trivial things”. Social adaptation difficulties consisted of four items, such as “I always feel that most people cannot be trusted” and “When I am depressed, I often do not want to talk to other people”. Each item was divided into 6 options, namely “lasting more than 3 months”, “lasting more than 2 months”, “lasting more than 1 month”, “lasting more than 2 weeks”, and “lasting more than 2 weeks”. “Lasted more than 2 weeks”, “Lasted more than 1 week”, and “No or lasted less than 1 week”. Subjects chose one item that best fit their reality, and each item was selected as a single choice. A score of 1 was recorded when the subject selected the first 3 items (“lasted more than 3 months”, “lasted more than 2 months”, “lasted more than 1 month”); when the subject selected the last 3 items (When the last 3 items were selected (“lasting more than 2 weeks”, “lasting more than 1 week”, “none or lasting less than 1 week”), a score of 0 was recorded. A score of ≥4, ≥1, and ≥2 for emotional symptoms, behavioral symptoms, and social adaptation difficulties was considered a positive result. The presence of psychological symptoms was defined as a score of ≥7 when the scores of the three dimensions were added together.

### Quality control

Our survey staff consisted of trained and qualified teachers and graduate students, 15 in total, divided into 3 groups to enter different provinces at the same time to conduct the survey. The schools were communicated with prior to the survey to agree on the testing time. A uniform instructional language was used to explain the purpose of the study and the requirements of the survey to the subjects before the survey.

The subjects were asked to fill in the questionnaire on the spot in the school classroom, and returned the questionnaire on the spot after completion. The questionnaire filling process took about 20–30 min.

### Statistical analyses

The expressions of duration of muscle exercise for different CHARACTERISTICS Chinese college students were expressed as percentages and mean ± standard deviation for categorical variables and mean ± standard deviation for continuous variables.

Comparisons of different duration of muscle exercise categorical variables were conducted by chi-square test. One-way ANOVA was used for the comparison of continuous variables with different durations of muscle exercise. Comparisons of the detection rates of emotional symptoms, behavioral symptoms, social adaptation difficulties, and psychological symptoms among college students with different durations of muscle exercise were also compared using the chi-square test. The comparison of the detection rates of emotional symptoms, behavioral symptoms, social adaptation difficulties, and psychological symptoms among college students with different durations of muscle exercise was also performed by chi-square test.

The association between duration of muscle exercise and psychological symptoms was analyzed by logistic regression analysis. Three models (Crude Model, Model 1, and Model 2) were used for logistic regression analysis. Crude Model did not adjust for confounding variables; Model 1 adjusted for age, father's education, mother's education, and SES; Model 2 adjusted for sleep duration, BMI, and MVPA on the basis of Model 1.

## Result

We surveyed 5,559 Chinese college students, of whom 2,414 (43.4%) were boys. The mean age of the subjects was (20.16 ± 1.04) years. The proportions of duration of muscle exercise in <30 min/d, 30–60 min/d, and >60 min/d were 75.0%, 20.4%, and 4.6%, respectively. The proportion of boys with duration of muscle exercise >60 min/d was higher than that of girls, and the difference was statistically significant when comparing the duration of muscle exercise between genders (*χ*^2^ of 88.602, *P* < 0.001). Our study also showed that the differences in duration of muscle exercise among Chinese college students were statistically significant (*P* < 0.05) in terms of father's education, mother's education, duration of sleep, and SES. Chinese college students with duration of muscle exercise of 30–60 min/d had the longest MVPA time of (31.14 ± 23.76) min, followed by those with >60 min/d at (22.99 ± 25.93) min (Table 1).

**Table 1:** Comparison of duration of muscle exercise among Chinese college students with different characteristics.

Characteristics	Duration of muscle exercise			Total	*χ*^2^*/F-*value	*P***-**value
	<30 min/d	30–60 min/d	>60 min/d			
Number of people	4,166 (75.0)	1,136 (20.4)	257 (4.6)	5,559		
Age (years)	20.15 ± 1.04	20.15 ± 1.03	20.28 ± 0.96	20.16 ± 1.04	1.860	0.156
Sex						
Boys	1,659 (39.8)	622 (54.8)	133 (51.8)	2,414 (43.4)	88.602	<0.001
Girls	2,507 (60.2)	514 (45.2)	124 (48.2)	3,154 (56.6)		
Father's education						
Primary school and below	1,207 (29.0)	304 (26.8)	68 (26.5)	1,579 (28.4)	14.713	0.005
Secondary and high school	2,635 (63.3)	730 (64.3)	153 (59.5)	3,518 (63.3)		
College degree and above	324 (7.8)	102 (9.0)	36 (14.0)	462 (8.3)		
Mother's education						
Primary school and below	1,956 (47.0)	491 (43.2)	106 (41.2)	2,553 (45.9)	21.178	<0.001
Secondary and high school	2,039 (48.9)	578 (50.9)	128 (49.8)	2,745 (49.4)		
College degree and above	171 (4.1)	67 (5.9)	23 (8.9)	261 (4.7)		
Duration of sleep						
≥8 h/d	1,018 (24.4)	423 (37.2)	106 (41.2)	1,547 (27.8)	96.964	<0.001
<8 h/d	3,148 (75.6)	713 (62.8)	151 (58.8)	4,012 (72.2)		
SES						
Low	679 (16.3)	154 (13.6)	37 (14.4)	870 (15.7)	16.378	0.003
Medium	2,946 (70.7)	795 (70.0)	173 (67.3)	3,914 (70.4)		
High	541 (13.0)	187 (16.5)	47 (18.3)	775 (13.9)		
BMI	23.40 ± 6.34	23.25 ± 6.13	22.75 ± 5.83	23.34 ± 6.28	1.448	0.235
MVPA	13.59 ± 14.67	31.14 ± 23.76	22.99 ± 25.93	17.61 ± 18.93	459.502	<0.001

Note: Descriptive statistics are presented as mean (standard deviation) and number (percentage) for continuous and categorical. Socioeconomic status (SES) Low (<15thSES), Medium (15-85thSES), High (>85thSES). BMI, body mass index; MVPA, Moderate and Vigorous Physical Activity.

Overall, the detection rate of psychological symptoms among Chinese college students was 9.0% (499/5559). The detection rate of psychological symptoms among boys students was 10.7% (258/2414) and 7.6% (241/3154) among girls students. The detection rates of emotional symptoms, behavioral symptoms, social adaptation difficulties, and psychological symptoms among Chinese college students with different durations of muscle exercise were compared. The differences were statistically significant (*χ*^2^-value 51.423, 62.986, 64.643, 55.468, *P* < 0.001). In terms of gender, there were statistically significant differences in the detection rates of emotional symptoms, behavioral symptoms, social adaptation difficulties, and psychological symptoms among boys students (*P *< 0.001), while there were no significant differences among girls students (Table 2). There were no significant differences among the girls (Table 2).

**Table 2:** Comparison of the detection rate of psychological symptoms among Chinese college students in duration of muscle exercise.

Psychological symptoms	duration of muscle exercise	*N*	Percentage	*χ*^2^-value	*P*-value
Boys					
Emotional symptoms	<30 min/d	249	15.0	69.357	<0.001
	30–60 min/d	24	3.9		
	>60 min/d	2	1.5		
Behavioral symptoms	<30 min/d	288	17.4	75.981	<0.001
	30–60 min/d	28	4.5		
	>60 min/d	5	3.8		
Social adaptation difficulties	<30 min/d	284	17.1	81.419	<0.001
	30–60 min/d	26	4.2		
	>60 min/d	3	2.3		
Psychological symptoms	<30 min/d	242	14.6	84.562	<0.001
	30–60 min/d	14	2.3		
	>60 min/d	2	1.5		
Girls					
Emotional symptoms	<30 min/d	240	9.6	6.168	0.046
	30–60 min/d	43	8.4		
	>60 min/d	4	3.2		
Behavioral symptoms	<30 min/d	276	11.0	9.179	0.01
	30–60 min/d	42	8.2		
	>60 min/d	5	4.0		
Social adaptation difficulties	<30 min/d	279	11.1	6.723	0.035
	30–60 min/d	41	8.0		
	>60 min/d	8	6.5		
Psychological symptoms	<30 min/d	200	8.0	3.958	0.138
	30–60 min/d	37	7.2		
	>60 min/d	4	3.2		
Total					
Emotional symptoms	<30 min/d	489	11.7	51.423	<0.001
	30–60 min/d	67	5.9		
	>60 min/d	6	2.3		
Behavioral symptoms	<30 min/d	564	13.5	62.986	<0.001
	30–60 min/d	70	6.2		
	>60 min/d	10	3.9		
Social adaptation difficulties	<30 min/d	563	13.5	64.643	<0.001
	30–60 min/d	67	5.9		
	>60 min/d	11	4.3		
Psychological symptoms	<30 min/d	442	10.6	55.468	<0.001
	30–60 min/d	51	4.5		
	>60 min/d	6	2.3		

Our findings showed that overall, after adjusting for relevant confounders (Model 2), duration of muscle exercise <30 min/d was positively associated with the occurrence of psychological symptoms in Chinese college students with duration of muscle exercise >60 min/d as the reference group (*OR*: 4.19, 95% *CI:* 1.82,9.61) (*P* < 0.001). There was a positive association between duration of muscle exercise <30 min/d and the occurrence of psychological symptoms (*OR*: 4.19, 95% *CI*: 1.82,9.61) (*P *< 0.001). There were positive associations between emotional symptoms (*OR*: 4.56, 95% *CI*: 1.99,10.44), behavioral symptoms (*OR*: 3.44, 95% *CI*: 1.79,6.60), social adaptation difficulties (*OR*: 3.04, 95% *CI*: 1.62,5.68) dimensions were also positively correlated (*P* < 0.01). In terms of gender, there was a positive association between duration of muscle exercise <30 min/d and the occurrence of psychological symptoms in Chinese college boys (*OR*: 9.02, 95% *CI*: 2.11,38.55) (*P *< 0.001). There was no association with psychological symptoms in girls. The association between duration of muscle exercise and psychological symptoms was higher in boys than in girls, i.e., the OR was higher in boys than in girls (Table 3).

**Table 3:** Logistic regression analysis of duration of muscle exercise and psychological symptoms among Chinese college students (*n* = 5,559).

Psychological Symptoms	Duration of Muscle Exercise	Odds Ratio (95% Confidence Interval)		
		Crude Model	Model 1	Model 2
Boys				
Emotional symptoms	>60 min/d	1.00 (Reference)	1.00 (Reference)	1.00 (Reference)
	30–60 min/d	2.63 (0.61,11.26)	2.41 (0.56,10.34)	2.08 (0.47,9.13)
	<30 min/d	11.57 (2.84,47.04)^b^	10.55 (2.58,43.08)^b^	8.36 (2.00,35.03)^b^
	*P*	<0.001	<0.001	<0.001
Behavioral symptoms	>60 min/d	1.00 (Reference)	1.00 (Reference)	1.00 (Reference)
	30–60 min/d	1.21 (0.46,3.19)	1.12 (0.42,2.96)	0.98 (0.36,2.66)
	<30 min/d	5.38 (2.18,13.26)^b^	4.98 (2.01,12.33)^a^	4.54 (1.76,11.68)^b^
	*P*	<0.001	<0.001	<0.001
Social adaptation difficulties	>60 min/d	1.00 (Reference)	1.00 (Reference)	1.00 (Reference)
	30–60 min/d	1.89 (0.56,6.34)	1.73 (0.51,5.81)	1.51 (0.44,5.23)
	<30 min/d	8.95 (2.83,28.31)^b^	8.20 (2.58,26.07)^b^	7.37 (2.24,24.24)^a^
	*P*	<0.001	<0.001	<0.001
Psychological symptoms	>60 min/d	1.00 (Reference)	1.00 (Reference)	1.00 (Reference)
	30–60 min/d	1.51 (0.34,6.72)	1.36 (0.31,6.10)	1.17 (0.25,5.42)
	<30 min/d	11.19 (2.75,45.50)^a^	10.11 (2.48,41.28)^a^	9.02 (2.11,38.55)^a^
	*P*	<0.001	<0.001	<0.001
Girls				
Emotional symptoms	>60 min/d	1.00 (Reference)	1.00 (Reference)	1.00 (Reference)
	30–60 min/d	2.74 (0.96,7.78)	2.91 (1.02,8.31)	2.95 (1.02,8.54)
	<30 min/d	3.18 (1.16,8.68)^a^	3.27 (1.19,8.97)^a^	2.85 (1.03,7.88)^a^
	*P*	<0.001	<0.001	<0.001
Behavioral symptoms	>60 min/d	1.00 (Reference)	1.00 (Reference)	1.00 (Reference)
	30–60 min/d	2.12 (0.82,5.47)	2.22 (0.86,5.74)	2.20 (0.84,5.77)
	<30 min/d	2.94 (1.19,7.27)^a^	3.00 (1.21,7.42)^a^	2.75 (1.1,6.83)^a^
	*P*	<0.001	<0.001	＜0.001
Social adaptation difficulties	>60 min/d	1.00 (Reference)	1.00 (Reference)	1.00 (Reference)
	30–60 min/d	1.26 (0.57,2.75)	1.33 (0.61,2.93)	1.29 (0.58,2.87)
	<30 min/d	1.82 (0.88,3.76)	1.86 (0.89,3.85)	1.68 (0.80,3.51)
	*P*	<0.001	<0.001	<0.001
Psychological symptoms	>60 min/d	1.00 (Reference)	1.00 (Reference)	1.00 (Reference)
	30–60 min/d	2.33 (0.81,6.66)	2.50 (0.87,7.18)	2.66 (0.91,7.75)
	<30 min/d	2.60 (0.95,7.12)	2.67 (0.97,7.34)	2.37 (0.86,6.55)
	*P*	<0.001	<0.001	<0.001
Total				
Emotional symptoms	>60 min/d	1.00 (Reference)	1.00 (Reference)	1.00 (Reference)
	30–60 min/d	2.62 (1.13,6.11)^a^	2.58 (1.10,6.02)^a^	2.33 (0.99,5.50)
	<30 min/d	5.56 (2.46,12.57)^b^	5.41 (2.39,12.25)^b^	4.56 (1.99,10.44)^b^
	*P*	<0.001	<0.001	<0.001
Behavioral symptoms	>60 min/d	1.00 (Reference)	1.00 (Reference)	1.00 (Reference)
	30–60 min/d	1.62 (0.82,3.19)	1.59 (0.81,3.13)	1.40 (0.70,2.78)
	<30 min/d	3.87 (2.04,7.32)^b^	3.76 (1.98,7.13)	3.44 (1.79,6.60)^b^
	*P*	<0.001	<0.001	<0.001
Social adaptation difficulties	>60 min/d	1.00 (Reference)	1.00 (Reference)	1.00 (Reference)
	30–60 min/d	1.40 (0.73,2.69)	1.37 (0.71,2.64)	1.20 (0.62,2.34)
	<30 min/d	3.50 (1.90,6.44)^b^	3.38 (1.83,6.25)^b^	3.04 (1.62,5.68)^a^
	*P*	<0.001	<0.001	<0.001
Psychological symptoms	>60 min/d	1.00 (Reference)	1.00 (Reference)	1.00 (Reference)
	30–60 min/d	1.97 (0.84,4.63)	1.93 (0.82,4.56)	1.68 (0.70,4.02)
	<30 min/d	4.97 (2.20,11.22)^b^	4.80 (2.12,10.87)	4.19 (1.82,9.61)^a^
	*P*	<0.001	<0.001	<0.001

Note: Model 1 adjusted for age, father's education, mother's education, and SES; Model 2 adjusted for duration of sleep, BMI, and MVPA on the basis of Model 1.

^a^ *P* < 0.05

^b^ *P* < 0.01.

## Discussion

Our study showed that the detection rate of psychological symptoms among Chinese college students was 9.0%, which was lower than the findings of depression symptoms among Chinese college students (43.77%) (22) and also lower than the findings of depression among Shanghai college students (35.68%) (23). There are two main reasons for the low results of this study. First, with the continuous reform and increasing investment in China's college education, colleges and universities pay particular attention to the health education of college students. Educational administrative departments require colleges and universities to set up student psychological education and counseling centers to monitor students' mental health status regularly. At the same time, the mental health education work has been increased to promote the development of college students' mental health for the better (24). Second, different studies suffer from inconsistencies in survey time, geography, and professional background of the group. Studies have shown that the level of health education among college students is influenced by their professional background, and the mental health status of medical students is better than that of non-medical students, which is related to the concern and attention to their own mental health brought by their medical background (25). It is also shown that there are differences in psychological symptoms among college students from different birth regions. For example, the aggressive behavior of college students in western China is higher than that in eastern China, which is closely related to the regional economic development and factors of academic and employment pressures (26). Third, the different evaluation criteria for psychological symptoms and the differences in the assessment tools used in different studies are also important reasons for the differences in different studies (27, 28). Overall, although the detection rate of psychological symptoms among Chinese college students is not high, there still exists a certain proportion of them, which should be given attention and importance, and active and strong measures should continue to be taken to prevent the occurrence of psychological symptoms.

In terms of gender, our findings showed that the detection rate of psychological symptoms was 10.7% for college boys and 7.6% for girls, which is inconsistent with the findings of related studies (29). Influenced by the new crown epidemic pandemic, the physical exercise time of college boys was strictly limited, resulting in more boys playing games and staying up all night. While girls pay more attention to their health and live a more regular life, the decline in physical activity, prolonged video screen time and reduced sleep quality among boys students will inevitably lead to an increase in the detection rate of psychological symptoms, resulting in a higher detection rate of psychological symptoms among boys students than girls students. In addition, the detection rate of boys' psychological symptoms is higher than that of girls, which is related to the increase of psychological symptoms caused by boys' temperament and irritability caused by the personality factors of boys and girls.

Our study divided the duration of muscle exercise among college students into three groups (<30 min/d, 30–60 min/d, and >60 min/d), which to some extent reflects the muscle strength level of college students. The study confirmed that there was a close positive correlation between duration of muscle exercise and muscle strength, indicating that there was an association between duration of muscle exercise and muscle strength (30). After adjusting for relevant confounders, logistic regression analysis showed that college students with shorter duration of muscle exercise (<30 min/d) had a higher risk of psychological the risk of symptoms was higher in college students with shorter duration of muscle exercise (<30 min/d) compared to those with higher duration of muscle exercise (>60 min/d) (*OR*: 4.19), and there was a negative association. Related studies have confirmed that muscle strength level or duration of muscle exercise is associated with lower psychological symptoms in children and adolescents, and that adherence to muscle strength exercise has a positive effect on reducing psychological symptoms (31). It has also been shown that children and adolescents who adhere to muscle strength exercises tend to have better levels of physical fitness and higher self-confidence in life and school, thus promoting healthy psychological development (32). Similarly, studies have also shown that children and adolescents with higher levels of mental health are more willing to participate in all types of physical activity, including muscle strength exercises, than those with lower levels of mental health (33). Studies have shown that children and adolescents with higher levels of muscle strength or who engage in regular muscle exercise also have higher levels of their own cardiorespiratory fitness, with the amount showing a positive correlation, while higher levels of cardiorespiratory fitness are associated with a lower incidence of psychological symptoms (34). These studies all explain the lower detection rate of psychological symptoms in college students with longer duration of muscle exercise. Our findings also suggest that the association between duration of muscle exercise and psychological symptoms is higher in Chinese college boys than in girls, i.e., the OR is higher in boys than in girls. The effect on psychological symptoms was more pronounced in boys because they participated in physical activity for longer and more frequently. On the contrary, college girls had less muscle strength and less floating, which led to a smaller effect on psychological symptoms.

Our study has some advantages. On the one hand, the sample size of the research survey is relatively large, involving three provinces in China, and the research results are representative to a certain extent. However, our study also has certain limitations. First, this study is a cross-sectional study, which can only analyze the association between duration of muscle exercise and psychological symptoms, but cannot understand the causal relationship between the two. Second, this study uses the duration of muscle exercise to reflect the muscle level of college students, and there is inevitably a certain deviation. In the future, a more comprehensive analysis of the relationship between muscle level and psychological symptoms should be combined with strength testing, frequency of muscle exercise, and intensity of muscle exercise. Third, although the covariates investigated in this study involve demographic factors, family status, physical activity, and sleep status, they are still limited, and the investigation of covariates should be further expanded in the future, such as sugar-sweetened beverage consumption, screen time and other factors. Fourth, in our study, body mass index and exercise were reported by the subjects, and there may be some deviations from the true values, which may have a certain impact on the results. Objective measurement methods should be used for evaluation in the future.

## Conclusions

In our study, duration of muscle exercise and psychological symptoms were investigated in 5,559 college students in China. After controlling for relevant confounders, the results of the analysis showed that there was a negative association between duration of muscle exercise and psychological symptoms among Chinese college students, i.e., a longer duration of muscle exercise was associated with a lower incidence of the longer duration of muscle exercise was associated with a lower incidence of psychological symptoms. Our research suggests that in the future we should work together to improve the duration of muscle movement and take effective measures to reduce the occurrence of psychological symptoms in college students. For example, increasing the duration of outdoor activities, conducting health education courses, and increasing muscle resistance exercise should be taken together to improve the physical and mental health of Chinese college students.

## Data Availability

The raw data supporting the conclusions of this article will be made available by the authors, without undue reservation.
